# Prevalence of Down Syndrome in Croatia in the Period from 2014 to 2024

**DOI:** 10.3390/medicina61111934

**Published:** 2025-10-28

**Authors:** Tomislav Benjak, Ana Vuljanić, Željka Draušnik, Irena Barišić, Zrinka Mach, Dinka Vuković, Tomislav Đidara, John Patrick Clarke, Gorka Vuletić

**Affiliations:** 1 Croatian Institute of Public Health, 10000 Zagreb, Croatia; tomislav.benjak@hzjz.hr (T.B.); ana.vuljanic@hzjz.hr (A.V.); irena.barisic@hzjz.hr (I.B.); tomislav.djidara@hzjz.hr (T.Đ.); 2 Croatian Society for Chronic Diseases, 10000 Zagreb, Croatia; zrinka.mach@gmail.com; 3 Croatian Down Syndrome Association, 10000 Zagreb, Croatia; hrdown.zajednica@gmail.com; 4 European Disability Forum, 1210 Brussels, Belgium; pat@patclarke.eu; 5 Faculty of Humanities and Social Sciences, Josip Juraj Strossmayer University of Osijek, 31000 Osijek, Croatia; gvuletic@ffos.hr

**Keywords:** Down syndrome prevalence, national registry of persons with disabilities, policies for persons with disabilities and Down syndrome

## Abstract

*Background and Objectives*: Individuals with Down syndrome (DS) represent a specific and vulnerable population requiring improvements in public health and social policies to ensure equal opportunities, longer life expectancy, and better quality of life. Accurate epidemiological and demographic indicators are essential for planning and evaluating interventions. This study aims to assess the prevalence of DS in Croatia from 2014 to 2024, analyzing demographic characteristics and regional distribution. A comparative analysis with international data and a review of national policies related to persons with disabilities and DS are also included. *Materials and Methods*: Data on the prevalence of DS were collected from the National Registry of Persons with Disabilities, where reporting individuals with DS is mandatory for the realization of legal rights. This ensures high data quality and representativeness. Prevalence per 1000 live births was calculated based on data from the national birth database and the registry. *Results*: The overall prevalence of DS in Croatia increased from 3.7 to 5.3 per 10,000 population during the observed period, while prevalence among live-born infants ranged from 1.1 to 1.5 per 1000. Males were slightly more represented (52.5%). The most common comorbidities included congenital heart defects. The mean age of individuals with DS was 28 years, with 12 individuals recorded as being older than 65 years and one individual aged 85. *Conclusions*: The DS prevalence in Croatia is comparable to data from European Union countries. The observed increase in prevalence and in the total number of individuals with disabilities highlights the need for continuous development and adaptation of national policies. As a signatory of the Convention on the Rights of Persons with Disabilities, Croatia is actively working to improve its legislative framework and support systems to ensure equal rights and enhance quality of life for individuals with DS.

## 1. Introduction

Down syndrome (DS) is the most common chromosomal disorder in humans, caused by the presence of a full or partial third copy of chromosome 21 [1] (first clinically described in 1866 by John Langdon Down, and linked to its chromosomal origin about a hundred years later by Jerome Lejeune [1,2]). DS, also known as trisomy 21, is the most common genetic cause of intellectual disabilities and developmental delays [3]. Individuals with DS face numerous health challenges that affect nearly all bodily and organ systems. They are predominantly of short stature with specific physical and cognitive characteristics [4,5].

Worldwide, one child with DS is born for every 700 live births [6]. Risk factors for trisomy 21 include parental age, particularly maternal age, but also paternal age, as well as previous pregnancies that resulted in chromosomal abnormalities [7]. The risk of having a child with DS ranges from 1 in 1400 for women giving birth between the ages of 20 and 24, 1 in 900 at age 30, 1 in 385 at age 35, 1 in 105 at age 40, and for women giving birth at age 45, the risk factor is 1 in 30 [8]. Over the past decades, advances in neonatal and cardiac care have significantly contributed to improving the survival rate of children with Down syndrome [9,10].

According to data from the European Surveillance of Congenital Anomalies (EUROCAT), DS accounts for approximately 8% of all congenital anomalies [11]. It is estimated that in Europe, from 2011 to 2015, 8031 children with DS were born, and without elective terminations, there would be 17,331 live births annually [12]. The increase in the prevalence of DS over the past few decades is mainly associated with the rising average age of mothers [11,13,14].

In the Republic of Croatia, as in most European Union countries, there is a noticeable trend of later pregnancies and increased maternal age [15], as well as older fathers [7]. This, based on the principles of human genetics and the incidence of DS, should lead to a higher occurrence and prevalence of DS compared to data from 20 or 30 years ago.

The aim of this study is to determine, based on data from the Croatian Registry of Persons with Disabilities for the period from 2014 to 2024, whether there has been an increase in the prevalence of this chromosomal disorder in Croatia, as reported in previously mentioned international studies in this field. Given the vulnerability of individuals with Down syndrome (DS), this study also seeks to describe the efforts of Croatian health, social, and other policy sectors aimed at promoting equal opportunities and improving the quality of life for persons with disabilities and their families.

The primary objective of this research is to estimate the prevalence of Down syndrome (DS) in Croatia during the period 2014–2024, both at the national level and disaggregated by counties, using data from the Registry of Persons with Disabilities.

The secondary objective is to describe the prevalence among live-born children and the demographic characteristics of registered DS cases.

## 2. Materials and Methods

Based on the Law, the Croatian Register of Persons with Disabilities from 2001, which is maintained by the Croatian Institute of Public Health (HZJZ), data on individuals whose disability is caused by Down syndrome (DS) are monitored [16]. The primary sources of data on DS as a cause of disability are findings and opinions regarding the severity and type of disability from assessment bodies. In Croatia, since 2015, disability assessments are conducted by a unified assessment body, the Institute for Assessment, Professional Rehabilitation, and Employment of Persons with Disabilities (ZOSI). Prior to that year, assessments were conducted by four different bodies, with the determination of disability for individuals with DS being handled by the Ministry of Social Policy and Youth, the Ministry of Science, Education and Sports, and the Croatian Pension Insurance Institute. Before 2015, assessment bodies did not uniformly designate DS as a cause of disability, with some committees only mentioning it in the anamnesis section, which significantly complicated the monitoring of this diagnosis. Additionally, monitoring was hindered by the fact that all data from previous assessment bodies, up to 2015, were submitted in paper format to the Register, necessitating their control and transcription into the database. During this control, special attention was paid to anamnesis textual data, where it was sometimes noted that an individual had DS, but this was not recorded by the committee among the ICD-10 codes for causes of disability. In such cases, the health professional from the Register assigned the ICD-10 code for DS (Q90) to the individual [17].

A small number of cases were also recorded during quality control where the assessment body mistakenly applied the ICD-10 code Q90. These were situations in which the commission should have used the code O90, as the disability assessment report submitted to the Register included anamnesis data indicating that the assessed female individual experienced complications during pregnancy. The commission intended to describe these complications using the ICD-10 code O90; however, due to an error, the diagnosis Q90 was recorded instead.

Such errors occurred because the diagnoses were transcribed into the printed version of the disability assessment report by an administrative staff member, and the medical assessor did not notice the incorrect entry of ICD-10 code Q90 instead of O90 at the time of signing the report. In these cases—and we emphasize that they were few—the Register did not actually record the diagnosis Q90 (Down syndrome), but rather O90, which denotes pregnancy-related complications in the assessed woman.

These efforts to improve data quality, which were not limited solely to Down syndrome but applied to all ICD-10 codes used in disability assessment reports, led the Register coordinator and the HZJZ to continuously advocate for improvements in legislation and IT systems within the assessment bodies. These improvements were aimed at ensuring more accurate application of ICD-10 codes. The need to enhance the assessment methodology—which included the aforementioned proposals from the HZJZ—ultimately resulted in the adoption of new Regulation on Assessment Methodologies from 2015 [18] which ensured easier monitoring of data on DS, as it was designated as a separate cause of disability with 100% impairment of the organism, which directed and facilitated the assessment bodies in marking this chromosomal disorder.

Significant progress was also made in the software solutions used by the disability assessment bodies, which now provide dropdown menus for diagnoses. Medical assessors no longer need to memorize ICD-10 codes, as was the case prior to 2015. Instead, it is now sufficient to enter the textual diagnosis, which the software automatically converts into the correct ICD-10 code. This significantly improved data coverage and the development of DS epidemiology. With the establishment of a unified assessment body and the adoption of the aforementioned Regulation, the HZJZ receives data on individuals with disabilities, including those with DS, through automatic delivery from the ZOSI assessment database.

For the purposes of displaying the prevalence and incidence of DS in this research, individuals with the ICD-10 code Q90 marked during the period from 2014 to 2024 were included. In the study, we use descriptive analysis. To calculate the prevalence of DS, or disability caused by it, in the Republic of Croatia overall and by counties, data from the Register of Persons with Disabilities and the official population estimates from the Croatian Bureau of Statistics for 2021 [19], as well as the International Classification of Diseases and Related Health Problems under the diagnosis code Q90 Down syndrome [17,20,21], were used. Data from the national birth database of the HZJZ regarding live births in Croatia by year were also utilized. For the analysis of the national policy related to persons with DS, the existing legislative acts related to persons with DS, i.e., to persons with disabilities, were analyzed. Joinpoint Trend Analysis Software Version 5.4.0 was used for analysis. The alpha level was set to 0.05 (5%).

## 3. Results

Based on data from the Registry of Persons with Disabilities and the annual population figures of the Republic of Croatia, an increase in the prevalence of Down syndrome (DS) can be observed over the period from 2014 to 2024—from 3.7 to 5.3 per 10,000 inhabitants (Table 1). Despite the observed increase in prevalence, there was no significant difference in rate over those 10 years (*p* > 0.05). Chi−square test for variation in annual rates across years was performed (χ^2^ = 0.8135; df = 9; *p*= 0.99).

Data from the Registry of Persons with Disabilities and the National Birth Database for the period 2014 to 2024 indicate a prevalence of Down syndrome among live births ranging from 1.1 to 1.5 per 1000 (Table 2). There was no significant difference in rate over the 11 years examined. Chi−square test for variation in annual rates across years was performed (χ^2^ = 0.1231; df = 10; *p* = 0.99).

We conducted trend analyses for two indicators related to Down syndrome in Croatia over the period 2014–2024. The first analysis assessed the number of persons with Down syndrome registered in the Registry of Persons with Disabilities, using the total Croatian population as the denominator. The second analysis examined the number of live-born children with Down syndrome, with the total number of live births in the same period serving as the denominator.

Both indicators were analyzed using Joinpoint regression analysis, a statistical method developed by the U.S. National Cancer Institute to identify points where a significant change in trend occurs. The method fits a series of joined straight lines on a logarithmic scale to the observed data and estimates annual percent changes (APC) for each segment. This approach allows detection of shifts in direction or magnitude of temporal trends, while also testing whether these changes are statistically significant.

Joinpoint regression was conducted using permutation tests and Monte Carlo methods with Bonferroni correction. Analysis was performed using Joinpoint Statistical Software with, given the number of time periods, maximum number of joinpoints set at 1. Logarithmic transformation was applied, with statistical significance for annual percent changes (APC) set at 0.05 level.

Data for 2019 were not available in the Registry of Persons with Disabilities. To maintain continuity in the time series, we imputed the value for 2019 by calculating the average of the adjacent years, 2018 and 2020.

**Figure 1:**
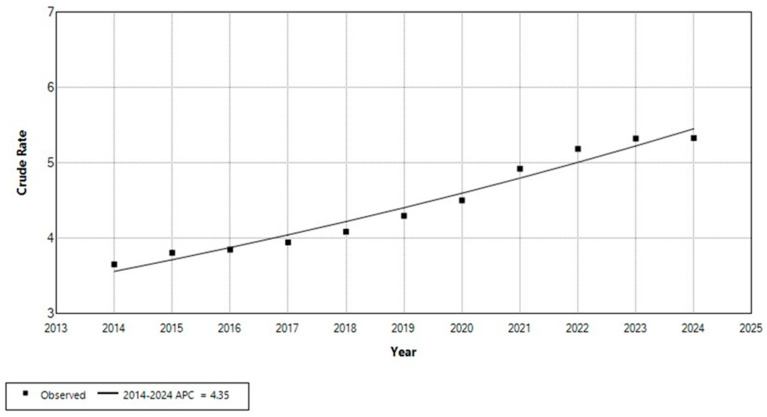
Trend in the number of persons with Down syndrome registered in the Registry of Persons with Disabilities per total Croatian population, 2014–2024 (rates per 10,000).

Joinpoint regression analysis of the number of persons with Down syndrome registered in the Registry of Persons with Disabilities, standardized to the total Croatian population for the period 2014–2024, showed a statistically significant increasing trend (Figure 1).

The estimated annual percent change (APC) was 4.35% (95% CI: 3.3 to 5.5, *p* < 0.05), indicating a steady rise in the proportion of individuals with Down syndrome recorded in the registry. In 2014, the crude rate of registered persons with Down syndrome was 3.66 per 10,000 population, increasing to 5.33 per 10,000 population in 2024.

An analysis of the age distribution among individuals with Down syndrome reveals that the majority fall within the 20–64-year age group (55.3%), while 44.1% are aged 0–19 years. Proportion of individuals with Down syndrome have also been recorded in the 65+ age group (0.6%) (Table 3).

**Figure 2:**
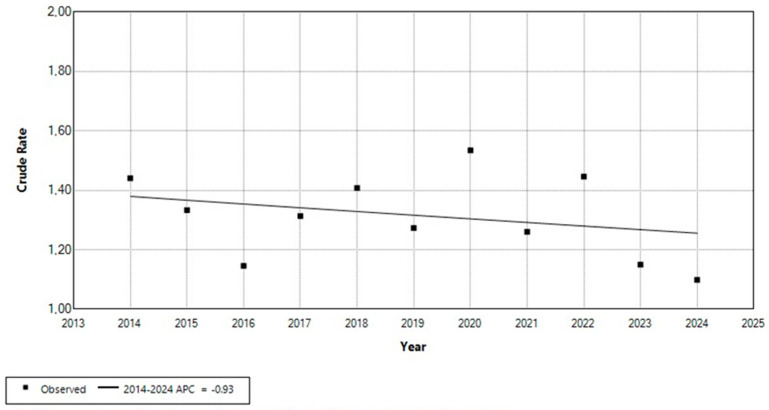
Trend in the number of live-born children with Down syndrome per total number of live births in Croatia, 2014–2024 (rate per 1000).

In contrast, analysis of the number of live-born children with Down syndrome relative to the total number of live births over the same period demonstrated a decreasing trend, with an estimated APC of −0.93% (95% CI: −4.1 to 2.2) (Figure 2). This change was not statistically significant (*p* > 0.05). In 2014, the crude rate of live-born children with Down syndrome was 1.44 per 1000 live births, compared to 1.10 per 1000 live births in 2024.

The analysis of National policies in the Republic of Croatia shows that the Republic of Croatia does not have legislative acts that refer exclusively to people with DS, however, as a cause of disability, DS is incorporated into various legislative acts that refer to people with disabilities, which is the basis for recognizing various rights for people with DS. For example, the aforementioned Register of Persons with Disabilities regularly collects and updates data on persons with disabilities, the Act on Professional Rehabilitation and Employment of Persons with Disabilities [20] defines the rights in the field of professional rehabilitation and employment of persons with disabilities, including persons with DS. The aforementioned Act is followed by the Ordinance on Professional Rehabilitation and Centers for Professional Rehabilitation of Persons with Disabilities [21,22], which defines in more detail the ways in which persons with disabilities acquire the right to professional rehabilitation. The Act on Inclusive Supplement [23] defines the ways in which persons with disabilities can obtain financial assistance, and the Act on Transport Benefits [24], as its name suggests, defines the rights of persons with disabilities in the field of transport, including the national and European disability cards.

## 4. Discussion

Croatia is one of the few countries worldwide with a comprehensive Registry of Persons with Disabilities, which also includes data on individuals with Down syndrome (DS). This Registry is maintained by the HZJZ in accordance with the Law on the Registry of Persons with Disabilities [16]. While questions about the completeness and relevance of such data for calculating the prevalence of this chromosomal disorder could be raised, the fact that persons with DS must undergo formal assessment to obtain their rights—and that, by law, these assessment results are mandatorily submitted to the Registry—indicates good data coverage. It can be assumed that very few persons with DS and their families have not needed to access social welfare, education, healthcare, transport, or other rights, and therefore remain unregistered. The coverage of persons with disabilities, including those with DS, in the Registry notably increased after 2015, following the establishment of a centralized assessment body and new legislation (Regulation on Assessment Methodologies [18]). This legislation, for the first time, explicitly listed chromosomal abnormalities—including DS—as causes of disability with 100% impairment.

Another factor contributing to increased coverage was the enactment of the Law on Traffic Privileges for Persons with Disabilities [24]. This law designated the Registry of Persons with Disabilities as the central proof of disability status. For obtaining transport-related rights, including issuance of the EU Mobility Card and parking permits, HZJZ issues a certificate confirming disability status. This certificate contains all necessary data enabling the Ministry of the Sea, Transport and Infrastructure to issue the documents according to legally defined criteria. Combined with data from HZJZ’s national birth database—which records the number of children born with DS—these sources provide a comprehensive and relevant dataset for determining DS prevalence in Croatia.

The prevalence of DS largely depends on previously mentioned risk factors, including the availability of prenatal diagnostics and the option to terminate pregnancies in the case of diagnosed congenital disorders. Countries with greater access to prenatal diagnostics and elective termination options—such as most European countries—typically report decreasing birth prevalence of DS and other congenital anomalies [12,25]. Differences between countries depend on early screening programs for DS and other malformations and the availability of elective termination [26].

According to the EUROCAT database, the prevalence of DS in Europe is 1.1 per 1000 live births [27]. When we compare the prevalence of DS in other European countries, it is relatively stable and ranges from a maximum of 2–3.4/1000 live births in Ireland and Malta, around 1.2/1000 in Great Britain, while in France the prevalence is the lowest (0.75/1000 live births) [27]. It is estimated that in 2015 there were approximately 419,000 individuals with DS living in Europe, and without elective terminations, this number would have been 574,000 [12]. A U.S. study estimated a DS prevalence of 8.27 per 10,000 population [28]. A ten-year prevalence trend study in Bosnia and Herzegovina, also based on estimates, found the prevalence of live-born children with trisomy 21 to be 9.6 per 10,000, remaining stable over time. This suggests that prenatal diagnosis had minimal impact on DS prevalence at birth, indicating a need for improved prenatal screening in developing countries [29].

Many DS prevalence studies rely on various estimation methodologies. The key strength of this ten-year Croatian study lies in the use of data directly from the Registry and analysis of recorded individuals. Since registration is linked to the realization of important rights, the authors consider the data coverage to be very high.

As of 27 July 2025, the Registry of Persons with Disabilities recorded 2177 individuals with DS in Croatia, corresponding to a prevalence of approximately 5 per 10,000 inhabitants. Between 40 and 50 children with DS are born annually, according to data from the Registry and the HZJZ birth database. In 2024, 35 children were born with DS (1.1 per 1000 live births), with the note that this figure will likely increase as Registry data are finalized within two to three years after birth, depending on when families request rights requiring assessment and registration.

These data suggest that Croatia’s DS prevalence among live births aligns with that of European Union countries, while the overall DS prevalence in the population shows an increasing trend consistent with international epidemiological research [11]. The observed increase in DS prevalence in Croatia between 2014 and 2024 year from 3.7 to 5.3 per 10,000 inhabitants between 2014 and 2024 was not statistically significant. This rise reflects better case ascertainment and a shrinking population, rather than an actual significant increase in the incidence of DS.

Data on the number of persons with DS by county indicate the highest counts in the City of Zagreb and Split-Dalmatia County, which together account for about 30% of all registered individuals with DS. No statistically significant differences in prevalence among counties were found. Sex distribution is nearly equal, with a slight male predominance (52.5% males vs. 47.5% females) [30], consistent with international data showing slightly higher DS frequency in males (ratio approximately 1.1:1) [31]. The majority of individuals fall into working-age (55%) and childhood (44%) categories. The average age of registered persons with DS is 28 years, with 12 individuals over 65 years and one aged 85. These findings align with evidence that the average life expectancy for persons with DS has increased beyond 25 years, with many living around 60 years on average [32,33]. The data refer only to those registered in National Registry of Persons with Disabilities. Unfortunately, the data on average life expectancy of persons with DS in Croatia are scarce. The prolonged lifespan largely results from enhanced healthcare quality, especially for leading comorbidities. In Croatia, congenital heart defects remain the most common comorbidity, affecting about 50% of children with DS [34]. Advances in pediatric cardiac surgery have been crucial in extending life expectancy among persons with DS [35,36].

Statistical data from the Registry have been continuously reported to the Croatian Down Syndrome Association from 2007, regularly every year. This umbrella organization coordinates national association activities and actively promotes inclusion, equality, and rights for individuals with this genetic condition [30]. The collected data form the critical basis for developing and improving public policies aimed at enhancing the quality of life for individuals with DS and their families. As a signatory to the United Nations Convention on the Rights of Persons with Disabilities [37], Croatia is obliged to regularly monitor and publish disability data to inform policies ensuring equal opportunities and improved quality of life for this vulnerable population and their families. This legal framework underpins a range of laws affecting persons with disabilities, including DS. In recent years, Croatia has enacted the Law on Inclusive Allowance [23], the Law on Traffic Privileges for Persons with Disabilities [24], and the Law on Professional Rehabilitation and Employment of Persons with Disabilities [20]. These laws also apply to individuals with DS. HZJZ data from the Registry contributed to financial impact analyses for the Inclusive Allowance Law and are directly involved in implementing laws on traffic privileges and employment through issuing status certificates. Moving forward, Registry data will support additional areas such as crisis management and evacuation planning, ensuring persons with disabilities and DS have equal access to emergency services.

Although data on persons with disabilities—including those with Down syndrome (DS)—are linked to the exercise of their rights based on disability status, there remains the possibility that not all individuals in Croatia are captured in the Registry. The fact that the Registry includes over 650,000 persons with disabilities (representing 17% of the Croatian population) indicates a high level of coverage; however, there is no precise method to verify the completeness of the data.

Several factors may have influenced this limitation, including the submission of data via postal mail prior to 2015, as well as the possibility that some individuals were never officially assessed for disability. The use of postal mail is highly susceptible to human error, representing a potential risk for incomplete data capture. While the likelihood that individuals—particularly those with DS, who typically require a high level of societal support—have never undergone assessment or sought their rights is relatively low, it cannot be entirely ruled out. This scenario may be more plausible in smaller communities, where families might refrain from applying for certain entitlements due to fear of stigma.

In recent years, the rights of persons with disabilities in Croatia have expanded, including financial support mechanisms. Therefore, it can be reasonably expected that data coverage within the Registry—for both persons with disabilities in general and those with DS—will continue to improve over time.

## 5. Conclusions

The prevalence of newborns with Down syndrome (DS) in the Republic of Croatia is comparable to that of most European Union countries, including those where prenatal diagnostics are widely available and pregnancy termination is legally permitted. Even this stable birth prevalence trend ultimately results in an increase in DS prevalence within the general population. An increase in population prevalence from 2014 to 2024 has also been observed in Croatia, as described in epidemiological research on DS [11]. According to the conclusions of this research, the rise in prevalence over recent decades is mainly associated with increasing maternal age. However, the data from this study indicate that the increase also stems from a growing number of registered individuals with DS and from depopulation. Prevalence is calculated as the ratio of the increasing number of registered DS cases (numerator) to the decreasing total population (denominator), which leads to the observed increase. Due to the rising number and longer lifespans of persons with disabilities, including those with DS, continuous efforts are needed to improve policies aimed at this especially vulnerable population. A higher-than-average prevalence of comorbidities or co-occurring health disorders in this population requires specific medical care, including adequate screening programs, tailored healthcare plans and guidelines, adapted to their specific needs, as well as optimal utilisation of both financial and healthcare resources [38].

As a signatory to the Convention on the Rights of Persons with Disabilities, the Republic of Croatia is actively enhancing legislation and conditions to promote equal opportunities and quality of life for persons with disabilities, including those with DS. The Registry serves as an important epidemiological tool for obtaining accurate data and forms the basis for securing the rights of this particularly vulnerable group.

## Figures and Tables

**Table 1:** Number of Individuals with Down Syndrome and Prevalence of Down Syndrome in the Period from 2014 to 2024 Based on Data from the Registry of Persons with Disabilities.

Year	Total Number of Registered Individuals with Down Syndrome in the Registry of Persons with Disabilities	Total Population of the Republic of Croatia by Year	Prevalence of Down Syndrome in the Total Population of the Republic of Croatia by Year/Per 10,000 Inhabitants	95% CI	
2014.	1550	4,238,389	3.7	3.475	3.839
2015.	1602	4,203,604	3.8	3.624	3.998
2016.	1608	4,174,349	3.9	3.664	4.040
2017.	1628	4,124,531	3.9	3.755	4.139
2018.	1672	4,087,843	4.1	3.894	4.286
2019.	1748	4,065,253	4.3	4.098	4.501
2020.	1824	4,047,680	4.5	4.299	4.713
2021.	1907	3,871,833	4.9	4.704	5.146
2022.	2000	3,855,641	5.2	4.96	5.415
2023.	2055	3,859,686	5.3	5.094	5.554
2024.	2058	3,859,686	5.3	5.102	5.562

**Table 2:** Number of Live-born Children with Down Syndrome and the Incidence of Down Syndrome per 1000 Live Births Based on Data from the Birth Database and the Registry of Persons with Disabilities of the Croatian Institute of Public Health.

Year	Number of Live-Born Children with Down Syndrome	Rate per 1000 Live-Born Children	95% CI	
2014.	57	1.4	1.067	1.815
2015.	50	1.3	0.964	1.703
2016.	43	1.1	0.803	1.488
2017.	48	1.3	0.942	1.685
2018.	52	1.4	1.025	1.790
2019.	46	1.3	0.905	1.641
2020.	55	1.5	1.129	1.94
2021.	46	1.3	0.896	1.624
2022.	49	1.4	1.041	1.851
2023.	37	1.2	0.78	1.521
2024.	35	1.1	0.735	1.463

**Table 3:** Number of People with Down Syndrome According to Age Group.

Age Group	Age 0–19	Age 20–64	Age 65+
Total	907	1139	12

## Data Availability

Restrictions apply to the availability of these data. Data were obtained from Croatian Institute of Public Health and are available at https://www.hzjz.hr/pristup-zdravstvenim-podacima/ (accessed on 1 August 2025 and 30 September 2025) with the permission of Croatian Institute of Public Health.
